# In Situ Evaluation of Epoxy Self-Healing Coating by Encapsulated Linseed Oil in Poly(Urea–Formaldehyde–Melamine) Microcapsules

**DOI:** 10.3390/ma18091906

**Published:** 2025-04-23

**Authors:** Lucas Henrique de Oliveira Souza, Michele Fedel, Fernando Cotting, Wagner Reis da Costa Campos

**Affiliations:** 1 Nuclear Technology Development Center, Belo Horizonte 31270-901, MG, Brazil; wrcc@cdtn.br; 2 Department of Industrial Engineering, University of Trento, Via Sommarive n. 9, 38123 Trento, Italy; michele.fedel@unitn.it; 3 Department of Chemical Engineering, Federal University of Minas Gerais, Belo Horizonte 31270-901, MG, Brazil; fernando@deq.ufmg.br

**Keywords:** self-healing, in situ characterization, electrochemical impedance spectroscopy, Raman spectroscopy, corrosion protection, polymeric microcapsules, linseed oil, organic coatings

## Abstract

The development of self-healing coatings represents a promising approach to enhance the durability of metal substrates exposed to corrosive environments, demanding thorough in situ investigations. In this study, poly(urea–formaldehyde–melamine) (PUF) microcapsules containing linseed oil (LO) were synthesized via in situ polymerization to act as healing agents in protective coatings. The microcapsules were characterized using scanning electron microscopy (SEM), optical microscopy (OM), Fourier-transform infrared spectroscopy (FTIR), and thermogravimetric analysis (TGA). The capsules exhibited a regular spherical morphology with an average diameter of 96 µm and an LO encapsulation efficiency of 81 wt%. TGA confirmed their thermal stability up to 200 °C, while FTIR verified the successful encapsulation of LO. For performance evaluation, 10 wt% of the microcapsules was incorporated into an epoxy matrix and applied to carbon steel. Corrosion resistance was evaluated using electrochemical impedance spectroscopy (EIS) in 0.1 mol/L of NaCl solution over 500 h. The coating with microcapsules exhibited a |Z|_0.01_ of 10^6^ Ω·cm^2^, higher than the 10^4^ Ω·cm^2^ observed for the coating without microcapsules, indicating improved barrier properties. Raman spectroscopy confirmed the auto-oxidation of LO at damaged areas, evidencing the self-healing mechanism. Although full barrier recovery was not achieved, the system effectively delayed corrosion progression.

## 1. Introduction

The corrosion of metals remains a critical global issue, often leading to severe structural failures through material degradation and damage. This phenomenon results in substantial economic losses across multiple industries, including the naval, construction, and aerospace sectors [1,2,3]. Globally, the direct annual costs associated with metal corrosion are estimated to range between USD 2.5 and 4.0 trillion [4], accounting for approximately 3% to 5% of national gross products—figures that exceed the total losses caused by all natural disasters and safety-related incidents combined [5,6,7,8]. To mitigate these effects and extend the operational lifespan of metallic structures and equipment, a variety of anticorrosive protection strategies are employed. These include material passivation, corrosion inhibitors, environmental modifications, and both cathodic and anodic protection techniques [9]. Among these, organic coatings are the most widely adopted due to their ability to effectively isolate metal surfaces from aggressive environments by preventing the ingress of water and oxygen [2,4,6,8,10,11,12,13,14]. Compared to other anticorrosion methods, organic coatings offer several advantages, including a broad applicability, ease of application, cost-effectiveness, and high protective performance [7,15,16]. However, the formation of cracks due to mechanical stress or environmental degradation significantly compromises their protective capability by creating pathways for corrosive species to reach the metal substrate [1,2,5,10,14,17,18]. Furthermore, these defects are often difficult to detect and repair using conventional techniques [2,5,10].

In this context, the development of self-healing smart coatings has emerged as a promising solution. These materials possess the ability to autonomously or externally repair microcracks, thereby enhancing the durability and reliability of polymer-based protective systems [2,5,15,19]. Among the various approaches to self-healing design, embedding organic or inorganic reservoirs within the coating matrix is particularly effective due to its versatility and ease of adaptation to different applications [1,11,20,21]. These reservoirs are typically loaded with film-forming agents that act as healing agents when released upon damage [4,21,22]. Microcapsules are the most commonly used reservoir structures due to their straightforward synthesis, strong encapsulation performance, and favorable mechanical properties [7,15]. Upon mechanical damage, these microcapsules rupture and release their core contents at the damaged site, where a polymeric film forms and restores the coating’s protective function [14,15,21,23,24,25,26,27,28]. Consequently, self-healing smart coatings represent a significant advancement in polymeric materials, contributing to an increased service life, operational efficiency, and environmental sustainability through reduced maintenance and repair costs [5,6,25,29,30].

Among the available microencapsulation techniques, in situ polymerization and interfacial polymerization are the most widely employed [1,7]. Common wall-forming polymers used in self-healing microcapsules include urea–formaldehyde resin [19], melamine–formaldehyde [22], polyurethane [2], and polystyrene [31]. Recently, the use of renewable raw materials in polymer resins and coatings has attracted growing interest due to their low cost, mechanical robustness, and environmental benefits [5]. Several curing agents have been explored for use in anticorrosion coatings, including isocyanate [29], epoxy resin [11], organic silane [31], and drying oil [32,33]. Among these, drying oils stand out for their sustainability, non-toxicity, availability, and cost-effectiveness. These oils form protective films through oxidative polymerization in air, eliminating the need for additional catalysts [10,12,19,25,30]. Specifically, linseed oil is a highly suitable candidate due to its high content of unsaturated esters—such as oleic, linoleic, and α-linolenic acids—and its affordability, environmental compatibility, water repellency, and ability to form flexible polymeric films upon exposure to atmospheric oxygen [2,10,17,25,34].

Despite the potential of self-healing polymeric materials, studies focusing on the in situ evaluation of healing processes—particularly those involving encapsulated drying oils—remain limited, as highlighted in recent works [3,23,35,36]. A comprehensive investigation into the self-healing performance of epoxy coatings containing encapsulated linseed oil is, therefore, essential to elucidate the underlying mechanisms and assess their true effectiveness in corrosion protection.

Therefore, this study aimed to evaluate the corrosion protection performance of a self-healing coating containing poly(urea–formaldehyde–melamine) microcapsules loaded with linseed oil, as well as to characterize, in situ, the linseed oil film formed in the defect region. For the morphological, chemical, and thermal characterization of the microcapsules, scanning electron microscopy (SEM), optical microscopy (OM), Fourier-transform infrared spectroscopy (FTIR), and thermogravimetric analysis (TGA) were employed. Electrochemical impedance spectroscopy (EIS) and OM were used to assess the self-healing effect of the coating. Additionally, micro-Raman spectroscopy was applied to analyze, in situ, the formation of the linseed oil film in the defect region.

## 2. Experimental Details

### 2.1. Materials

Urea and formaldehyde (37 wt% aqueous solution) were purchased from Synth. Ammonium chloride was purchased from Fmaia, resorcinol was purchased from Neon, and melamine and Arabic gum were purchased from Sigma-Aldrich. Linseed oil was obtained from Tintas Mococa. All of the chemicals were used without further purification.

### 2.2. Microcapsule Synthesis

Poly(urea–formaldehyde–melamine) microcapsules were synthesized via an in situ polymerization technique, following a methodology similar to that described in Patent WO2014032130A1 [37]. In a 400 mL beaker, a mixture consisting of 150 g of water, 20.0 g of linseed oil, 2.5 g of urea, 0.25 g of ammonium chloride, 0.25 g of resorcinol, 0.55 g of Arabic gum, and 0.25 g of melamine was prepared. This mixture was subjected to mechanical stirring at 800 rpm. After one hour of agitation, the pH was adjusted to 3.5 using a 10% hydrochloric acid solution. Subsequently, 6.4 g of formaldehyde was introduced into the system. The reaction was maintained at a constant temperature of 55 °C for 4 h. At the end of the synthesis, the microcapsules obtained were isolated through vacuum filtration and then dried in an oven at 35 °C for a period of 24 h.

### 2.3. Microcapsule Characterization

The microcapsules were analyzed by FTIR-ATR using a Vertex 70v (Bruker, Billerica, MA, USA, EUA) coupled with ATR platinum diamond (4 cm^−1^ resolution, 128 scans, and 4000–400 cm^−1^ wavenumber range).

Thermogravimetric analysis (TGA) was performed on a DTG-60H analyzer (Shimadzu, Kyoto, Japan) from 25 to 700 °C with a heating rate of 10 °C min^−1^. Measurements were performed in a nitrogen atmosphere with a flow rate of 50 mL min^−1^.

A Sigma VP (Carl Zeiss, Cambridge, UK) scanning electron microscope (SEM) equipped with an EDS detector and a DM 4500P (Leica, Wetzlar, Germany) polarization microscope was employed to observe the morphology of the microcapsules.

The size distribution of microcapsules was calculated by image processing in manual mode using ImageJ software (https://imagej.net/ij/, NIH Image, Bethesda, MD, USA).

The oil content in the microcapsules was quantified using a solvent extraction method, as previously described in the literature [11,38]. A 1.0 g sample of microcapsule was ground in a mortar with 5 mL of xylene. Following maceration, the mixture was placed in an ultrasonic bath for 5 min and then vacuum-filtered. The residue collected on the filter paper was washed again with xylene, macerated, and re-filtered to ensure the complete extraction of the encapsulated oil. The solid material remaining on the filter (polymeric matrix) was dried in an oven at 60 °C for 48 h. After drying, the retained material was weighed in order to determine the proportion of encapsulated oil and polymer in the sample.

% encapsulated material = 100 − [(mass retained material)/(total mass of sample) × 100](1)

This procedure was carried out in triplicate to ensure the reliability of the results.

### 2.4. Preparation of Epoxy-Coated Mild Steel Samples

AISI 1020 steel samples were used as substrates for the application of the coatings. The 1020 steel samples were blasted with a glass microsphere until a roughness profile of 10 ± 2 µm was achieved. The roughness was analyzed using a SJ-301 roughness meter (Mitutoyo, Kawasaki, Japan). After the blast step, the samples were cleaned with a dry airflow.

The microcapsules were added to an epoxy coating with 97% solids that met the PETROBRAS N-2680 standard [39]. A single-layer coating system with a thickness of approximately 250 µm was applied. The amount of microcapsules incorporated into the paint was 10% in relation to the mass of total solids, according to the paint specification. The coating layer was applied using a 2″ brush and wet thickness control was performed using a comb-type wet layer thickness gauge with a range of 25–370 Microns.

### 2.5. Characterization of Epoxy-Coated Mild Steel Samples

The thickness of the coating was measured by a digital coating thickness gauge (DIGIMESS TT210, São Paulo, Brazil). The average dry film thickness of the coating was approximately 150.60 μm ± 19.80 µm.

The coated panels were observed under a Jeol (Akishima, Japan) JSM-IT300 scanning electron microscope (SEM) to investigate the distribution of microcapsules within the coating matrix from the cross-section.

### 2.6. Electrochemical Impedance Spectroscopy

An Autolab PGSTAT302N potentiostat galvanostat (Metrohm, Herisau, Switzerland) was used in a three-electrode configuration, where a coated steel panel with an exposed area of 9.25 cm^2^ acted as the working electrode. Platinum and Ag/AgCl/3.5 M KCl electrodes were chosen as the counter and the reference electrodes, respectively.

The area was delimited by attaching a plastic tube with neutral curing silicone adhesive to the coated specimens. In the EIS measurements, the electrolyte used was an aqueous solution of 3.5 wt% NaCl for the intact coating and 0.1 mol/L NaCl for the scratched coating. As the real area of metal exposed to the electrolyte is critically significant for results obtained through EIS, it is very critical to perform reproducible defects in order to achieve confident results. In this context, EIS measurements were conducted on coated panels, both with and without induced defects. Under intact coating conditions, the aim was to assess whether the presence of microcapsules disrupted the performance of the coating system. Conversely, in the presence of induced defects, the objective was to evaluate the self-healing capability of the samples containing microcapsules.

Using a scalpel, 1.5 cm scratch-shaped defects were created in the samples to evaluate the self-healing effects and stimulus responsiveness of the coatings containing microcapsules.

Electrochemical impedance spectroscopy (EIS) measurements were conducted 14 days after the application of the coating layer to ensure the complete curing of the epoxy system. For undamaged samples, impedance spectra were recorded over a frequency range from 100 kHz to 10 mHz. Due to the high resistivity associated with the intact coatings, a sinusoidal excitation signal of 20 mV was applied at the open-circuit potential (OCP), and data were collected at ten points per frequency decade to ensure accuracy. In the case of damaged coatings, the same frequency range was employed, using a 10 mV sinusoidal perturbation amplitude at the OCP, with an identical data acquisition rate of ten points per frequency decade. Prior to each measurement, an OCP time of 1500 s was performed to stabilize the samples.

The electrochemical impedance spectroscopy (EIS) tests on damaged samples were conducted with the following two distinct approaches: first, with the damaged samples immediately exposed to the corrosive environment after defect creation, and second, with the damaged samples exposed to ambient air for 5 days following defect creation. This interval allowed sufficient time for the linseed oil released from the microcapsule core to cure and crosslink upon contact with atmospheric oxygen before exposure to the corrosive environment. For the scratched samples, EIS measurements were performed for 500 h of immersion in the electrolyte. In contrast, the intact samples were evaluated over a period of up to 1000 h of immersion. During the EIS tests, the progress of corrosion in the damaged samples was recorded by a camera attached to the optical microscope. Data analysis was conducted by the software ZView2 using classical equivalent electrical circuits.

### 2.7. Raman Spectroscopy in Situ Characterization

Raman spectra were recorded with an alpha300 RA-Raman imaging microscope (WITec, Working, UK) equipped with a continuous-wave laser with emission at 532 nm, with a grating of 600 lines mm^−1^. A power of 2.0 mW was used to analyze the samples. All the measurements were performed with a 50× objective (NA = 0.50). The spectra were obtained with an integration time of 30 s.

## 3. Results and Discussion

### 3.1. Physicochemical Characterization of the Pigments

#### 3.1.1. Fourier Transform Infrared Spectroscopy

**Figure 1:**
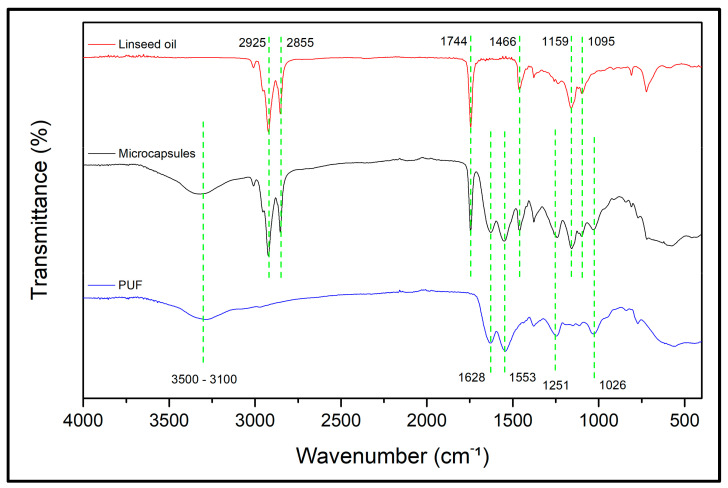
FTIR spectra of poly(urea–formaldehyde–melamine) (PUF) resin, linseed oil, and microcapsules containing core material.

The FTIR spectra obtained for linseed oil, the synthesized microcapsules, and the pure poly(urea–formaldehyde–melamine) (PUF) polymer are presented in Figure 1.

Analyzing the spectra of PUF and the synthesized microcapsules, it is observed that both exhibit similar characteristic peaks, such as the stretching vibrations of O–H and N–H between 3500 and 3100 cm^−1^ [40], the stretching vibration of C=O at 1628 cm^−1^, and the stretching vibration of N–H at 1553 cm^−1^ [1,41,42], as well as absorption peaks at 1251 cm^−1^, corresponding to the stretching of C–N, and at 1026 cm^−1^, associated with the stretching vibration of C–O from CH_2_OH groups formed during reactions between urea and formaldehyde monomers [35,36].

In the spectra of linseed oil and the microcapsules, absorption peaks are identified at 2925 cm^−1^, corresponding to the asymmetric stretching vibrations of =C–H, and at 2855 cm^−1^, corresponding to the symmetric stretching vibrations of C–H in (C–H)CH_2_ groups [1,17,32,35,41]. The characteristic peaks at 1744 cm^−1^ and 1159 cm^−1^ are attributed to the stretching vibrations of ester C=O and C–O present in linseed oil [1,17,25,32,35,42], while the absorption peak at 1466 cm^−1^ corresponds to the angular deformation of C–H bonds in CH_2_ groups [1,35].

Since the FTIR spectrum of the synthesized microcapsules exhibits characteristic peaks of both pure PUF and linseed oil, it is established that the linseed oil was successfully encapsulated by the PUF resin.

#### 3.1.2. Thermogravimetric Analysis

**Figure 2:**
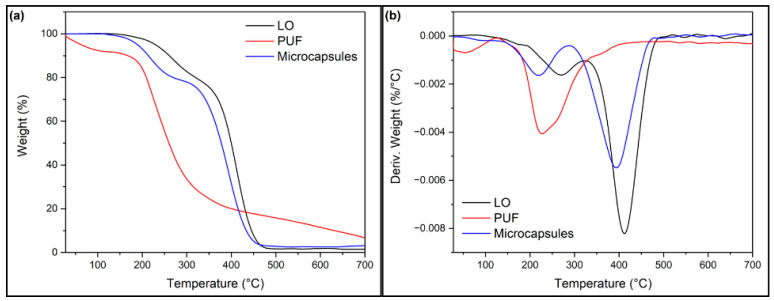
(**a**) Thermogravimetric (TGA) and (**b**) differential thermogravimetric (DTG) of poly(urea–formaldehyde–melamine) (PUF) resin, linseed oil (LO), and microcapsules containing core material.

Thermal analyses were conducted to further characterize the microcapsules. Figure 2 presents the thermogravimetric (TGA) and derivative thermogravimetric (DTG) curves of the microcapsules, as well as those of pure poly(urea–formaldehyde–melamine) (PUF) resin and linseed oil. Based on the TGA and DTG curves, it was observed that the pure PUF exhibited the following two stages of mass loss: the first occurring in the temperature range from 30 °C to 160 °C, mainly attributed to the elimination of free formaldehyde and the removal of residual trapped water [2,32,33,42], and the second between 180 °C and 370 °C. Pure linseed oil began its thermal decomposition at approximately 250 °C, with degradation occurring up to 470 °C, as evidenced by its weight loss curve [32,40,41]. Figure 2 shows that the microcapsules exhibited two distinct stages of thermal degradation. The first stage, occurring in the temperature range of 185–320 °C, corresponds to the degradation of the PUF shell, resulting in a mass loss of approximately 22% [1,43]. The second stage, between 355 °C and 485 °C, is related to the degradation of the encapsulated linseed oil [32,42]. These findings confirm that the synthesized microcapsules were composed of PUF and linseed oil [1,42]. The results indicate that the prepared microcapsules exhibited a good thermal stability, as their degradation began at approximately 200 °C [40,42,43]. Furthermore, according to the weight loss curve of the microcapsules, approximately 78% of the material remained at 340 °C, suggesting that the fraction of encapsulated linseed oil in the microcapsules was approximately 78% by weight. This value is consistent with the results obtained through the solvent extraction method [1].

#### 3.1.3. Particle Size and Linseed Oil Content of the Microcapsules

**Figure 3:**
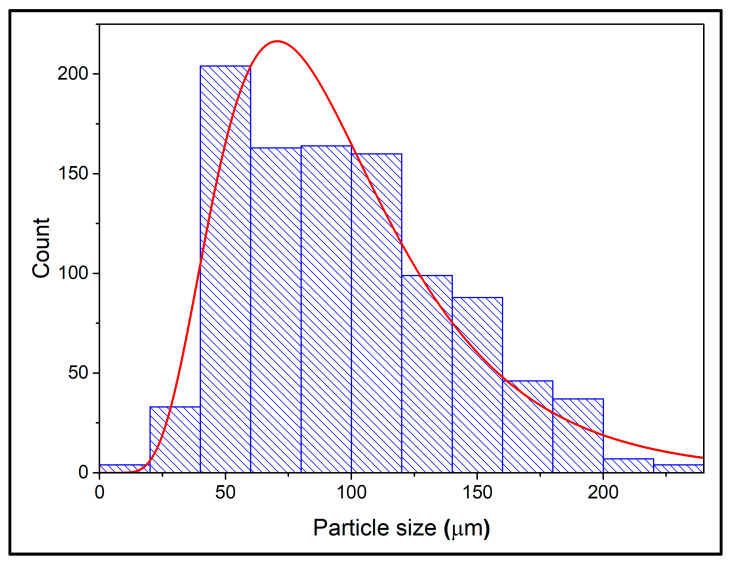
Histogram of the particle size distribution of poly(urea–formaldehyde–melamine) microcapsules containing the linseed oil.

To determine the size distribution and diameter of the poly(urea–formaldehyde–melamine) microcapsules containing linseed oil, images obtained from scanning electron microscopy were analyzed using the ImageJ software. A total of 1000 microcapsules were examined, and the results were plotted in a histogram to assess the particle size distribution. The histogram representing the size distribution of the microcapsules is presented in Figure 3.

Analysis of the histogram in Figure 3 reveals that the poly(urea–formaldehyde–melamine) microcapsules containing linseed oil exhibited a particle size distribution ranging from 20 μm to 220 μm, with an average diameter of 96 μm. Most of the particles fell within the size range from 40 μm to 110 μm. The observed particle sizes are consistent with values reported in the literature by other researchers [19,41,43,44].

The amount of linseed oil encapsulated in the PUF microcapsules determined by the extraction method was 81 ± 2 wt%, indicating that the encapsulation was effective. Previous research reported the encapsulated core content is in the range of 72–82 wt% for linseed oil [2,3,19,23,36].

#### 3.1.4. Microcapsule Morphology

**Figure 4:**
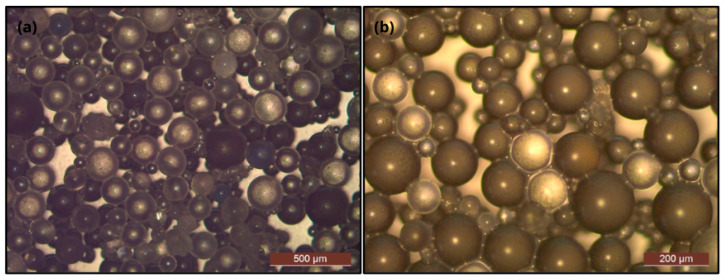
Optical microscope images of microcapsules synthesized, (**a**) 50× and (**b**) 100×.

Morphological analysis plays a fundamental role in verifying the microencapsulation process. Optical microscopy was employed to observe the morphology of the synthesized microcapsules. As illustrated in the images presented in Figure 4, the microcapsules exhibited a spherical morphology characterized by a smooth surface and the absence of aggregation. This spherical conformation is essential for the efficient storage of the healing agent and its homogeneous dispersion within the coating [42]. The optical microscopy observations also revealed the presence of diffraction rings, evidenced by dark circles around the microcapsules and bright points within them. According to optical theory, such diffraction rings arise due to differences in the refractive indices between the core and the shell of microcapsules, indicating the successful encapsulation of the core by the wall material [1,43,44].

**Figure 5:**
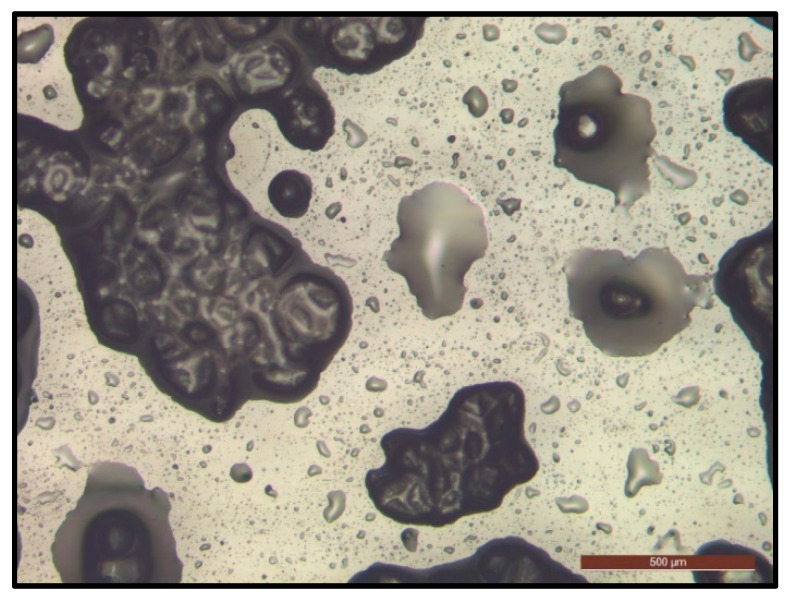
Optical microscopy images after microcapsules being pressed.

However, mere morphological inspection of the microcapsules via optical microscopy does not conclusively ensure the effective confinement of the healing agent within the polymeric core or its controlled release on demand. In this context, the release of liquid material upon the rupture of the microcapsules was used as a simplified approach to verify the presence of the healing agent in the microcapsule core [31]. For this purpose, the microcapsules were deposited onto a glass slide and subjected to compression using a spatula. The optical microscopy analysis of the ruptured microcapsules (Figure 5) demonstrated the significant release of liquid material, indicating that linseed oil was successfully encapsulated. These results confirm the formation of a core–shell structure with efficient encapsulation.

**Figure 6:**
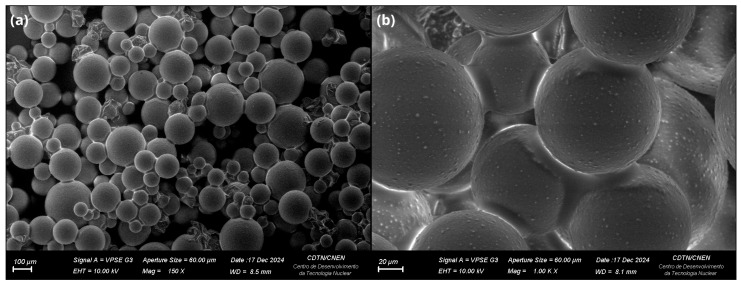
SEM images of microcapsules with rough surfaces, (**a**) 150× and (**b**) 1000×.

The morphology of the microcapsules was also characterized by SEM. As observed in the SEM images (Figure 6), the microcapsules exhibited a spherical shape and their surface was covered with solid material, resulting in surface roughness [28,40,45]. The spherical shape of the particles promoted better dispersion of the particles in the epoxy resin [15,46], while the roughness ensured proper adhesion to the coating matrix, which facilitated the rupture of the microcapsules due to the stress generated in the scratched area [1,3,32,42,44]. The rough surface of the capsules resulted from the accumulation of higher-molecular-weight polymeric nanoparticles adhering to the capsule surface [7,40,42,43]. The aggregations observed among the microcapsules were likely due to the presence of unwashed linseed oil on the capsule surface [15].

#### 3.1.5. Scanning Electron Microscopy of Cross-Section of Epoxy Coating

**Figure 7:**
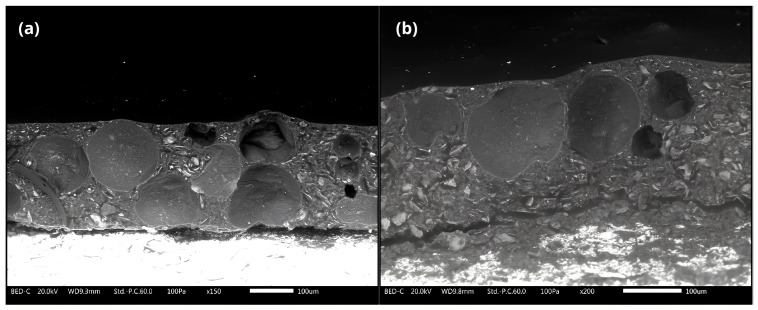
SEM image from cross-section of embedded (10 wt.%) microcapsules in epoxy coating, (**a**) 150× and (**b**) 200×.

The homogeneous distribution of microcapsules within the polymeric matrix of self-healing coatings is a highly relevant and impactful issue. An inadequate dispersion of microcapsules in the coating can result in regions without self-healing capacity [21]. To evaluate the distribution of microcapsules in the epoxy coating, scanning electron microscopy (SEM) images of the cross-section were employed. Figure 7 displays these images, revealing a uniform dispersion of microcapsules throughout the coating and a concentration gradient towards the surface, which can be attributed to the lower density of the microcapsules compared to the epoxy resin [38]. Furthermore, the images confirm that the microcapsules retained their spherical structure within the coating, without damage during the application process, indicating that they exhibited adequate mechanical resistance [42].

### 3.2. Electrochemical Impedance Spectroscopy of the Intact Coatings

**Figure 8:**
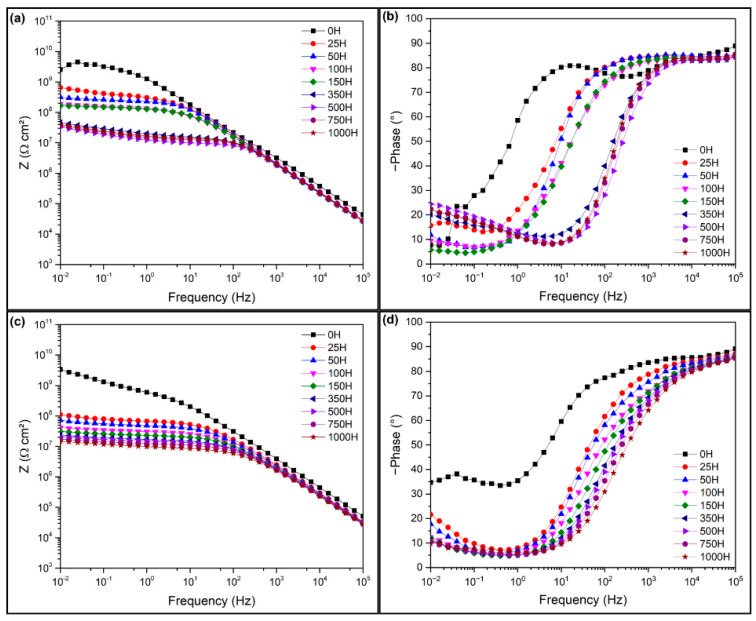
EIS diagrams of the intact coatings during 1000 h in 3.5 wt% NaCl. (**a**,**b**) Pure epoxy resin coating (EP0) and (**c**,**d**) composite coating with 10 wt% microcapsules (EP10).

The pure epoxy resin coating (EP0) and the composite coating with 10 wt% microcapsules (EP10) were immersed in a 3.5 wt% NaCl solution and studied using electrochemical impedance spectroscopy (EIS) at regular intervals over a period of 1000 h. Figure 8 presents the Bode plots (log |Z| vs. log f) of the coatings and the trend of low-frequency impedance (|Z|_0.01_) variation over 1000 h. The |Z|_0.01_ value of EP0 decreased from an initial 2.54 × 10^9^ Ω⋅cm^2^ to 1.65 × 10^8^ Ω⋅cm^2^ within 150 h (Figure 8a). After 1000 h of immersion, the |Z|_0.01_ value further decreased by two orders of magnitude to 4.01 × 10^7^ Ω⋅cm^2^.

In the case of EP10, the |Z|_0.01_ value decreased from an initial 3.35 × 10^9^ Ω⋅cm^2^ to 1.12 × 10^8^ Ω⋅cm^2^ within 25 h (Figure 8c). This one-order-of-magnitude decline in EP10 within 25 h may be attributed to the addition of microcapsules, which affected the crosslinking degree of the coating system, thereby reducing the impedance value of the coating. This effect may have occurred because the presence of microcapsules larger than 30 µm decreased the barrier property of the coating, creating preferential pathways for water and ion diffusion within the coating film [7,10,11]. However, after 1000 h of exposure, the |Z|_0.01_ value of EP10 decreased by two orders of magnitude relative to its initial value, reaching 1.45 × 10^7^ Ω⋅cm^2^. This indicates that, after 1000 h of exposure, the impedance modulus decay was similar for both coatings, demonstrating that the barrier property was not significantly affected by adding microcapsules.

**Figure 9:**
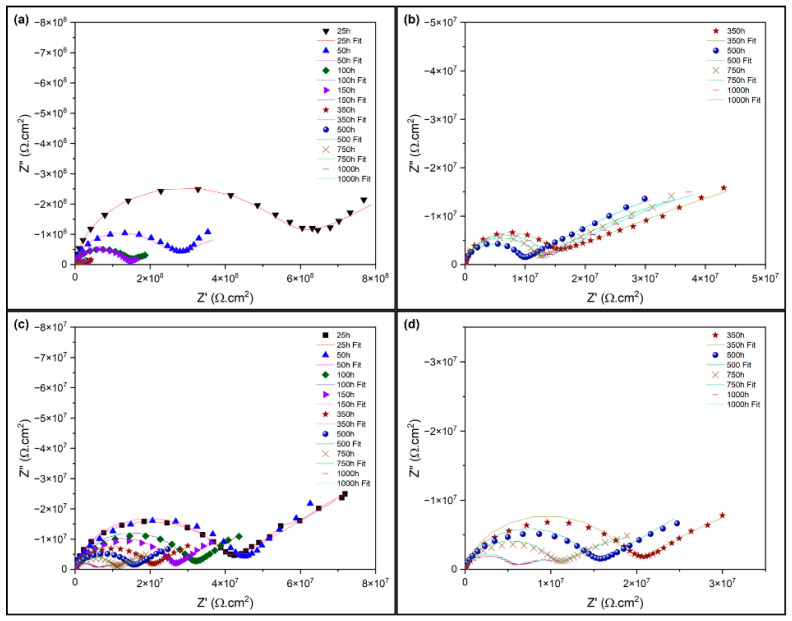
Nyquist plots for intact coating in the immersion of 3.5 wt% NaCl solution. (**a**) EP0; (**b**) Zoom EP0; (**c**) EP10; and (**d**) Zoom EP10.

Figure 9 presents Nyquist diagrams corresponding to the intact coating samples. It can be observed that both the EP0 and EP10 coatings exhibited a predominantly capacitive behavior, which indicates an excellent barrier property of these coatings [10]. Additionally, the Nyquist diagrams reveal the presence of a second time constant in the low-frequency region, a phenomenon also noticeable in the Bode plots (phase angle vs. log f).

**Figure 10:**
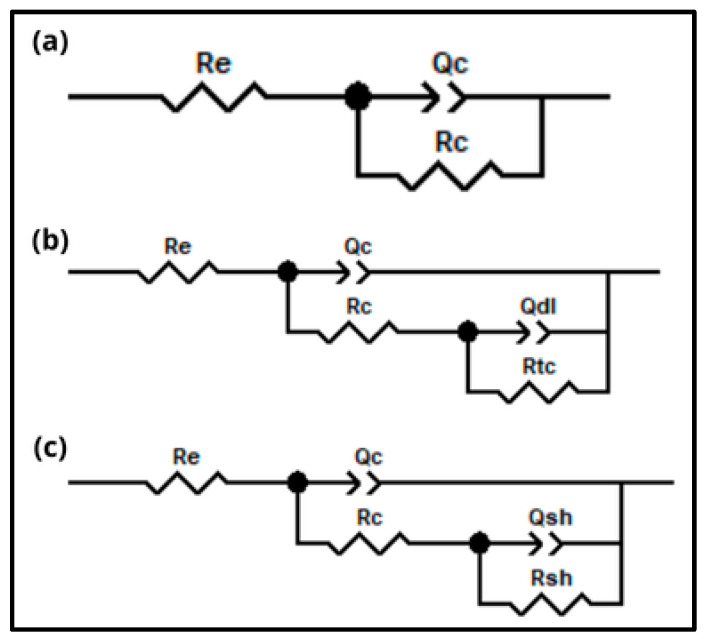
Equivalent circuits used for fitting the EIS spectra: (**a**) one-time constant, (**b**) two-time constants, and (**c**) two-time constants self-healing.

For the quantitative analysis of the spectra obtained in the EIS tests, the equivalent electrical circuits shown in Figure 10 were used. To more accurately represent the surface heterogeneities typical of coated metals, a constant phase element (CPE) was used instead of an ideal capacitance [11,16]. In the equivalent circuits, R_e_ represents the electrolyte resistance between the reference electrode and the working electrode. Due to the high resistance of the analyzed coating systems, the frequency range used in the experiments did not allow for the direct extraction of the R_e_ value from the electrochemical impedance spectroscopy (EIS) diagrams, leading to the decision to fix this parameter. The coating resistance is denoted as R_c_, while Q_c_ refers to the CPE associated with the coating capacitance. Additionally, R_tc_ represents the charge transfer resistance, Q_dl_ corresponds to the CPE related to the double-layer capacitance, Q_sh_ corresponds to the CPE related to the self-healing capacitance, and R_sh_ represents the self-healing resistance.

The equivalent electrical circuit shown in Figure 10b was employed to fit the EIS results obtained for the intact coatings. According to the data obtained by fitting the EIS data, it was observed that both analyzed systems, EP0 and EP10, exhibited low Q_c_ values and high values of the n_c_ parameter, characteristics that indicate a predominantly capacitive behavior of the coating. This behavior highlights the excellent barrier property provided by the coatings under study [10]. The presence of the second time constant in the low-frequency region can be attributed to the exposure of the coated metallic substrate to an aqueous medium, which resulted in a high double-layer capacitance value (Q_dl_). A high Q_dl_ value is mainly associated with changes in the dielectric properties of a coating, attributed to water uptake, as water possesses a significantly higher dielectric constant [47,48]. Water ingress into the coating likely occurred due to the presence of microporosity within the coating and the formation of preferential pathways resulting from the incorporation of microcapsules. These factors favor the diffusion of water and ions to the metallic substrate, thus deviating the system from purely capacitive behavior. The effects of water and ion diffusion to the substrate are corroborated by the high Q_dl_ values and the low n_dl_ values observed in Table 1. The variation in the n parameter was directly related to the degree of interaction between diffusing polar species and the polymer matrix; the greater this interaction, the lower the n value [48]. The low n_dl_ values suggest that the electrochemical processes at the metallic substrate occurred under diffusional control, primarily driven by the migration of water through the coating layer [49]. It is important to emphasize that the observed diffusional behavior did not precisely correspond to the ideal Warburg impedance model, as the n_dl_ value deviated from the theoretical value of 0.5 [50]. Therefore, it was observed that the studied coatings exhibited excellent barrier properties but were also susceptible to water and ion diffusion due to the existence of microporosities and the formation of preferential pathways within the coating [11].

Due to the limited number of experimental points in the low-frequency range, the second time constant could not be accurately fitted during the first two days of immersion. Although a low-frequency relaxation process was identified in the raw EIS data sets, the obtained fit was not reliable. Table 1 summarizes the fitting results for each type of intact coating sample throughout the exposure period.

### 3.3. Electrochemical Impedance Spectroscopy of the Damaged Coatings

**Figure 11:**
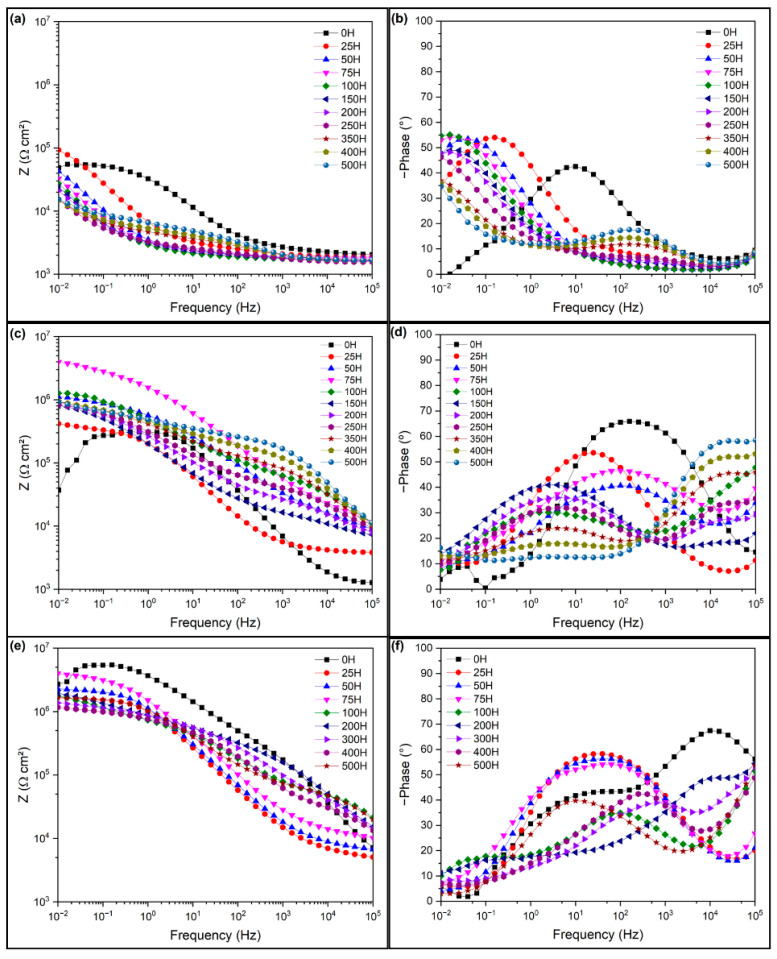
EIS diagrams of the damaged coatings during 500 h in 0.1 mol/L NaCl. (**a**,**b**) Pure epoxy resin coating (EP0); (**c**,**d**) composite coating with 10 wt% microcapsules immediate immersion (EP10-I); and (**e**,**f**) composite coating with 10 wt% microcapsules five-day curing (EP10-D).

The previously scratched surface was immersed in a 0.1 M NaCl solution, and its self-healing capacity was analyzed using electrochemical impedance spectroscopy (EIS). The effectiveness of the self-healing process of the coatings was evaluated by the following two methods: one involving the immediate immersion of the samples after the creation of the defect (EP10-I) and another referring to the immersion of the samples after a five-day curing period with linseed oil on the defect (EP10-D). Figure 11 presents the Bode diagrams obtained at different time intervals after immersion.

As shown in Figure 11a, the EP0 coating presented a value of |Z|_0.01_ of 1.53 × 10^4^ Ω⋅cm^2^ after 500 h of immersion. Compared to the intact EP0 coating, the damaged version of EP0 exhibited a three-orders-of-magnitude reduction in impedance, with a value of |Z|_0.01_ considerably lower than that of the intact EP0. This phenomenon can be attributed to the inability of the pure epoxy coating to retain its anticorrosive properties in the presence of microcracks. On the other hand, Figure 11e shows that the EP10-D coating presented a value of |Z|_0.01_ of 1.64 × 10^6^ Ω⋅cm^2^ after 500 h of immersion. Compared to the results obtained for the intact EP10 coating, the impedance modulus of the scratched EP10-D only suffered a reduction of one order of magnitude. Notably, the impedance modulus of the damaged EP10-D at 0.01 Hz was very close to that of the intact coating, indicating that the microcrack in EP10-D was effectively repaired by the released linseed oil.

A similar result was obtained for the scratched EP10-I coating (Figure 11c), where it was observed that after 500 h of immersion, the value of |Z|_0.01_ for EP10-I was 9.21 × 10^5^ Ω⋅cm^2^, indicating a reduction of approximately one order of magnitude when compared to the intact EP10 coating. Therefore, when comparing the scratched EP10-D and EP10-I coatings with the scratched EP0, it was observed that the values of |Z|_0.01_ for the scratched EP10-D and EP10-I remained higher than that of the scratched EP0 after 500 h of immersion. This result suggests that the released linseed oil acted as a film-forming agent, protecting the microcracked region from corrosive processes. The increase in total impedance observed in the samples containing microcapsules was related to the formation of a protective film in the damaged area due to the release of the encapsulated linseed oil, thus promoting the self-healing effect [11].

However, the scratched self-healing coating exhibited a loss of its barrier properties when immersed in the 0.1 M NaCl solution. For both the scratched EP10-D and EP10-I, a second time constant was identified after 75 h of immersion in the Bode plot (phase angle × log f), Figure 11d,f, suggesting the formation of double-layer capacitance. Given that this capacitance resulted from the presence of the electrolyte below the coating layer, it is evident that the scratched self-healing coating lost its barrier properties, allowing for the diffusion of water and ions to the metal substrate after 75 h of immersion [10]. This behavior was expected, since the corrosion resistance of linseed oil is relatively lower than that of synthetic resins, such as epoxy [19].

The results demonstrate that mechanical damage to the coating caused the rupture of the microcapsules along the affected area, leading to the release of encapsulated linseed oil in the damaged region and resulting in the formation of a protective film. The self-healing effect provided by the microcapsules was evidenced by the increase in the impedance modulus of the coatings analyzed. It was observed that the EP10-D sample presented a superior performance compared to the EP10-I sample, exhibiting an impedance modulus one order of magnitude higher. This result can be attributed to the fact that linseed oil is a drying oil, curing in the presence of atmospheric oxygen, ultimately forming a solid film [35], which provides more efficient protection. However, it was found that this protection is temporary, gradually decreasing over time due to the degradation of the linseed oil.

**Figure 12:**
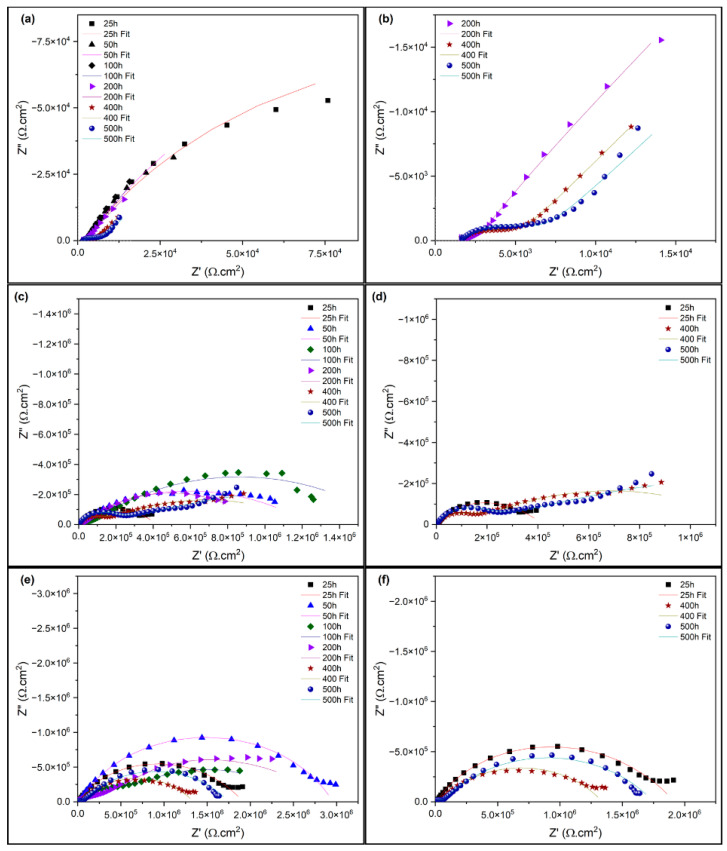
Nyquist plots for damaged coating in the immersion of 0.1 mol/L NaCl solution. (**a**) EP0; (**b**) Zoom EP0; (**c**) EP10-I (**d**) Zoom EP10-I; (**e**) EP10-D; and (**f**) Zoom EP10-D.

Figure 12 displays the Nyquist diagrams corresponding to the coating samples after the introduction of the defect. Analysis of these diagrams reveals that the EP10-I and EP10-D samples exhibited two time constants, indicating the presence of the self-healing film in the compromised region. To fit the data obtained from the damaged samples without microcapsules, the equivalent electrical circuits illustrated in Figure 10 were used.

Based on the values obtained, it was observed that the EP0 sample, after the introduction of the defect, presented a high capacitance value and a low n value for the first layer, Q_c_ and n_c_, respectively, when compared with the values obtained for the intact coating. This phenomenon can be attributed to the degradation of the protective properties of the coating due to the defect, which facilitated the exposure of the carbon steel substrate to the corrosive environment, causing the coating to no longer present a purely capacitive characteristic [11]. During the first 100 h of exposure, the damaged EP0 coating exhibited behavior consistent with the equivalent circuit represented in Figure 10a, characterized by a single time constant. After 100 h of exposure, the damaged EP0 coating was best fitted by the equivalent circuit shown in Figure 10b due to the emergence of a second time constant. This second time constant was associated with the formation of corrosion products that acted as a porous barrier, as evidenced by the high Q_dl_ values and low n_dl_ values, suggesting a diffusion process of the corrosive medium through the corrosion products until reaching the substrate surface.

The electrochemical response of the coatings containing 10 wt.% microcapsules was fitted using the equivalent circuit presented in Figure 10c. Comparing the capacitance (Q_c_) and n (n_c_) values presented by the coating containing microcapsules after the defect was created with the values presented for the intact coating, we observed an increase in the Q_c_ value and a reduction in the n_c_ value after the defect was created. This occurred because when the coating broke, it lost its protective property, causing the coating to no longer present a purely capacitive characteristic [11]. However, analysis of the data for the damaged EP10-I and EP10-D samples reveals the presence of the self-healing film in the affected region, as indicated by the increase in R_sh_, the reduction in Q_sh_ values, and the increase in n_sh_ when compared to the Q_dl_, R_tc_, and n_dl_ values of the EP0 sample. After 100 h of exposure, an increase in Q_sh_ values and a decrease in n_sh_ were observed, indicating a decline in the barrier efficiency of the linseed-oil-based film over time. The n_sh_ values approaching 0.5 suggest that a diffusion process occurred over time, allowing the electrolyte solution to penetrate the metal interface through discontinuities in the healing layer, initiating the corrosion process [2,49,50].

Therefore, it can be concluded that the coating containing microcapsules demonstrated a self-healing capability and effectively delayed corrosion progression after coating failure. Moreover, the results obtained from electrochemical impedance spectroscopy corroborate the findings from the immersion test, confirming the efficacy of the self-healing mechanism. Table 2 summarizes the fitting results for each type of damaged coating sample throughout the exposure period.

### 3.4. Corrosion Resistance by Immersion Study

**Figure 13:**
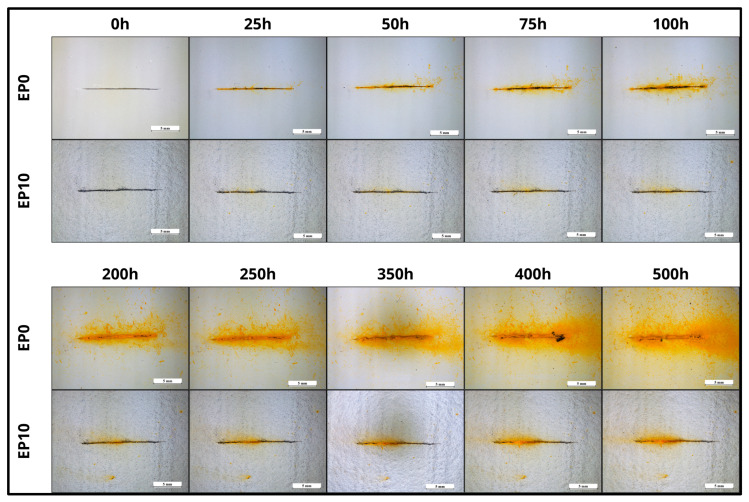
Immersion studies performance without microcapsules (EP0) and with microcapsules coatings (EP10) at different exposure periods.

In parallel with the analyses of electrochemical impedance spectroscopy (EIS), optical microscopy images of the pure epoxy resin coating (EP0) and the coating containing 10% microcapsules (EP10) were examined to evaluate the curing efficiency and the extent of corrosion protection provided by the self-healing coating. Figure 13 presents the images obtained after immersing the coatings in a corrosive solution (1 mol/L NaCl) for a period of 500 h.

The EP0 coating exhibited visible signs of oxidation within 24 h of immersion, showing severe corrosion after 100 h, particularly in the scribed region, with rust spreading over the substrate surface [23,30,51]. In contrast, the EP10 coating did not show significant rust formation during the first 100 h of immersion. After 500 h, only slight rust formation was observed in the scribed region [30,45]. Isolated corrosion points could be identified on the EP10 coating outside the defect area, which can be attributed to the reduced coating thickness relative to the average diameter of the microcapsules. This discrepancy promoted the formation of preferential pathways for the ingress of aggressive species in areas where microcapsule clusters occurred [31].

The superior performance of the EP10 coating in terms of corrosion resistance can be explained by the formation of a protective film in the defect region, resulting from the oxidative polymerization of the healing agent (linseed oil) released from the ruptured microcapsules. This film prevented the diffusion of saline ions, oxygen, and moisture, thereby reducing the corrosion process on the metallic substrate [3,5,23,51].

Therefore, based on the analyses conducted from the optical microscopy images, it can be concluded that this self-healing coating shows potential as a temporary solution to protect metallic substrates against corrosion, providing additional time for damage identification and repair [19].

### 3.5. In Situ Characterization by Raman Spectroscopy

**Figure 14:**
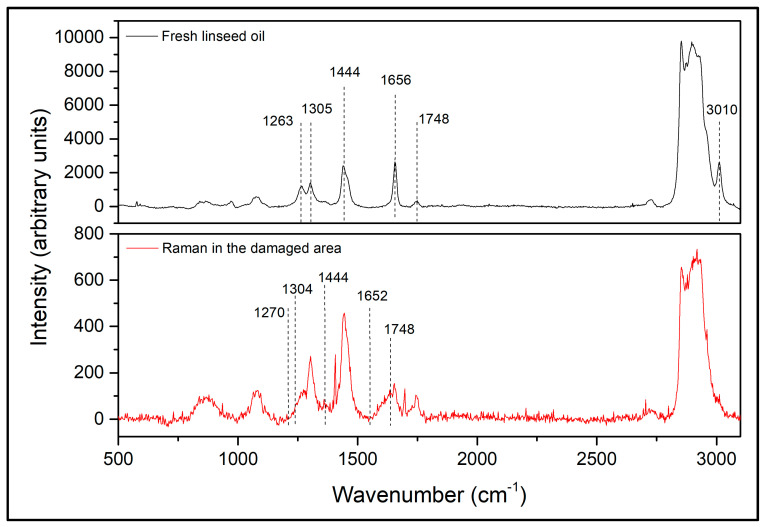
Raman spectrum of fresh linseed oil and the scratched region.

Figure 14 presents the Raman spectrum in the defect region of the self-healing coating. The obtained Raman spectrum exhibited characteristic bands of the fatty acids present in linseed oil, such as the in-plane CH deformation vibration of the cis –CH=CH– unit at 1270 cm^−1^ [52,53,54]; the two CH deformation vibrations specific to the saturated CH_2_ group, τ(CH_2_) and δ(CH_2_), around 1304 cm^−1^ and 1444 cm^−1^ [53,54], respectively; and the stretching vibration of the cis C=C bond in cis –CH=CH– at 1652 cm^−1^ [55,56]. The band at 1748 cm^−1^ can be attributed to the C=O stretching vibration of esters [53,54,55]. Comparing the Raman spectra of the fresh oil with those obtained in the defect region, a characteristic reduction in the intensity of the peaks at 1270 cm^−1^ and 1652 cm^−1^ was observed, together with the broadening of the carbonyl stretching peak at 1748 cm^−1^ [55,56]. These findings corroborate the occurrence of the oxidative drying process, indicating that the double bonds underwent degradation. Additionally, the degradation of the C=C double bond was evidenced by the absence of the band at 3010 cm^−1^, attributed to the C=C–H vibration, in the Raman spectra of the damaged region [55,56]. Thus, the results suggest that mechanical damage to the coating led to the rupture of microcapsules within the defect, promoting the release of the encapsulated linseed oil. Upon exposure to the environment, this oil underwent auto-oxidation, forming a film over the compromised area.

## 4. Conclusions

Poly(urea–formaldehyde–melamine) microcapsules containing linseed oil were successfully synthesized by in situ polymerization. SEM analysis showed that the synthesized microcapsules exhibited a spherical morphology with dimensions in the range of 20–220 μm. By correlating the infrared spectra of the wall material and the core material of the microcapsules, a satisfactory encapsulation of the oils by the wall material was confirmed. The thermogravimetric analysis confirmed the good thermal stability of the microcapsules and that theyed present a core content amount of approximately 80 wt%. Loading the coating system with self-healing microcapsules caused a small decrease in the total impedance of the coating system. However, electrochemical impedance spectroscopy and optical microscopy verified the self-healing effect in the samples containing microcapsules. The epoxy coating loaded with 10 wt% microcapsules showed an improved anticorrosive performance compared to the coating without microcapsules. However, the results show that linseed oil alone was only partially effective as a curing agent. The in-situ evaluation of the coating by Raman spectroscopy confirmed that the linseed oil formed a film in the defect region due to the auto-oxidation process of the linseed oil when in contact with oxygen, confirming the self-healing characteristic of the coating containing the microcapsules.

## Figures and Tables

**Table 1:** Fitting results of EIS data for intact coating samples. R_e_, R_c_, and R_tc_ (Ω·cm^2^); Q_c_ and Q_dl_ (nF·cm^2^/Sα^−1^); and n (α).

Immersion Time	Sample	Parameters						
		R_e_	Q_c_	n_c_	R_c_	Q_dl_	n_dl_	R_tc_
100 h	EP0	2000	3.23 × 10^−10^	0.8801	1.01 × 10^8^	5.90 × 10^−8^	0.5514	7.47 × 10^7^
	EP10	2000	2.55 × 10^−10^	0.8639	2.96 × 10^7^	2.09 × 10^−7^	0.4465	1.97 × 10^8^
150 h	EP0	2000	1.78 × 10^−10^	0.9286	6.76 × 10^7^	7.70 × 10^−9^	0.5275	7.27 × 10^7^
	EP10	2000	2.34 × 10^−10^	0.8752	2.31 × 10^7^	2.38 × 10^−7^	0.4238	6.70 × 10^7^
350 h	EP0	2000	1.63 × 10^−10^	0.9361	2.04 × 10^7^	4.21 × 10^−8^	0.3765	1.40 × 10^8^
	EP10	2000	2.50 × 10^−10^	0.8717	1.71 × 10^7^	2.40 × 10^−7^	0.4323	5.44 × 10^7^
500 h	EP0	2000	1.53 × 10^−10^	0.9395	2.01 × 10^7^	6.72 × 10^−8^	0.4533	1.10 × 10^8^
	EP10	2000	2.48 × 10^−10^	0.8731	1.38 × 10^7^	2.46 × 10^−7^	0.4276	3.78 × 10^7^
750 h	EP0	2000	1.21 × 10^−10^	0.9508	1.13 × 10^7^	1.02 × 10^−7^	0.4447	1.01 × 10^8^
	EP10	2000	2.80 × 10^−10^	0.8664	1.06 × 10^7^	2.76 × 10^−7^	0.4465	2.17 × 10^7^
1000 h	EP0	2000	1.14 × 10^−10^	0.9556	1.23 × 10^7^	9.07 × 10^−8^	0.4315	1.13 × 10^8^
	EP10	2000	2.57 × 10^−10^	0.8790	6.62 × 10^6^	2.37 × 10^−7^	0.4192	9.50 × 10^6^

**Table 2:** Fitting results of EIS data for damaged coating samples. R_e_, R_c_, R_sh_, and R_tc_ (Ω·cm^2^); Q_c_, Q_sh_, and Q_dl_ (nF·cm^2^/Sα^−1^); and n (α).

Immersion Time	Sample	Parameters									
		R_e_	Q_c_	n_c_	R_c_	Q_sh_	n_sh_	R_sh_	Q_dl_	n_dl_	R_tc_
25 h	EP0	2000	-	-	-	-	-	-	4.27 × 10^−5^	0.6223	2.75 × 10^5^
	EP10-I	1000	5.79 × 10^−7^	0.4164	4576	3.42 × 10^−7^	0.7536	7.60 × 10^5^	-	-	-
	EP10-D	2000	8.49 × 10^−8^	0.5288	11,505	7.48 × 10^−8^	0.7629	1.91 × 10^6^	-	-	-
50 h	EP0	2000	-	-	-	-	-	-	1.15 × 10^−4^	0.6254	3.72 × 10^5^
	EP10-I	1000	3.97 × 10^−7^	0.4394	22,372	4.33 × 10^−8^	0.7477	6.26 × 10^6^	-	-	-
	EP10-D	2000	4.42 × 10^−9^	0.7119	12,252	9.04 × 10^−8^	0.7187	2.94 × 10^6^	-	-	-
100 h	EP0	2000	-	-	-	-	-	-	2.13 × 10^−4^	0.6092	1.52 × 10^5^
	EP10-I	1000	1.03 × 10^−8^	0.7494	214,474	4.90 × 10^−7^	0.4179	5.04 × 10^6^	-	-	-
	EP10-D	2000	1.09 × 10^−7^	0.6553	638,130	1.27 × 10^−6^	0.5488	1.85 × 10^6^	-	-	-
200 h	EP0	2000	5.25 × 10^−5^	0.4317	1961	-	-	-	1.70 × 10^−4^	0.5719	1.54 × 10^8^
	EP10-I	1000	2.41 × 10^−7^	0.4583	35,667	1.01 × 10^−6^	0.5509	1.65 × 10^6^	-	-	-
	EP10-D	2000	3.01 × 10^−7^	0.3935	927,800	3.95 × 10^−7^	0.7629	2.12 × 10^6^	-	-	-
400 h	EP0	2000	1.82 × 10^−5^	0.5012	3521	-	-	-	2.52 × 10^−4^	0.5039	7.35 × 10^4^
	EP10-I	1000	6.37 × 10^−8^	0.5885	129,935	1.46 × 10^−6^	0.4360	1.25 × 10^6^	-	-	-
	EP10-D	2000	3.76 × 10^−10^	0.8849	33,789	1.92 × 10^−7^	0.6114	1.30 × 10^6^	-	-	-
500 h	EP0	2000	1.30 × 10^−5^	0.5380	4306	-	-	-	2.57 × 10^−4^	0.5541	1.10 × 10^5^
	EP10-I	1000	2.72 × 10^−8^	0.6472	169,330	1.97 × 10^−6^	0.5046	2.22 × 10^6^	-	-	-
	EP10-D	2000	1.45 × 10^−9^	0.7808	56,978	1.84 × 10^−7^	0.6085	1.69 × 10^6^	-	-	-

## Data Availability

The original contributions presented in this study are included in the article. Further inquiries can be directed to the corresponding author.

## Abbreviations

The following abbreviations are used in this manuscript:

SEM	Scanning Electron Microscopy
OM	Optical Microscopy
FTIR	Fourier-Transform Infrared Spectroscopy
TGA	Thermogravimetric Analysis
EIS	Electrochemical Impedance Spectroscopy
OCP	Open-Circuit Potential
PUF	Poly(Urea–Formaldehyde–Melamine)
DTG	Derivative Thermogravimetric
LO	Linseed Oil
EP0	Pure Epoxy Resin Coating
EP10	Composite Coating With 10 wt% Microcapsules
CPE	Constant Phase Element
